# PF-4708671 Activates AMPK Independently of p70S6K1 Inhibition

**DOI:** 10.1371/journal.pone.0107364

**Published:** 2014-09-09

**Authors:** Gilad W. Vainer, Ann Saada, Juliane Kania-Almog, Adir Amartely, Jacob Bar-Tana, Rachel Hertz

**Affiliations:** 1 Department of Pathology, Tel-Aviv Sourasky Medical Center, Tel-Aviv, Israel; 2 Monique and Jacques Roboh Department of Genetic Research, and Department of Genetics and Metabolic Diseases, Hadassah-Hebrew University Medical Center, Jerusalem, Israel; 3 Department of Pathology, Hadassah-Hebrew University Medical Center, Jerusalem, Israel; 4 Human Nutrition and Metabolism, Hebrew University Medical School, Jerusalem, Israel; Mayo Clinic, United States of America

## Abstract

The P70 ribosomal protein S6 kinase 1 (P70S6K1) is activated by the mammalian target of rapamycin (mTORC1) and regulates proliferation, growth, and metabolism. PF-4708671 is a novel, cell-permeable, has been proposed to be a highly specific inhibitor of p70S6K1. It is used in micromolar concentration range to dissect signaling pathways downstream of mTORC1 and to study the function of p70S6K1. Here we show that PF-4708671 induces AMP-activated protein kinase (AMPK) phosphorylation and activation in immortalized mouse embryonic fibroblasts (MEF) independently of p70S6K1, due to specific inhibition of mitochondrial respiratory chain Complex I.

## Introduction

P70 ribosomal protein S6 kinases (p70S6K) 1 & 2 (S6K1, S6k2) are two isoforms of the AGC kinase (protein kinase A/protein kinase G/protein kinase C) family. The AGC kinase family consists of about 60 members that share a conserved catalytic kinase domain [1], making it difficult to find a specific inhibitor for each individual member of this family.

p70S6K isoforms are activated by the phosphorylation of Thr389 catalyzed by the rapamycin-sensitive mammalian TOR complex 1 (mTORC1) [2]–[5]. Activated p70S6K1 is considered a key kinase in body metabolism. Activated p70S6Ks phosphorylates rpS6 on five clustered residues, allowing the initiation of protein translation [6]. Also, mitochondria number and size is increased in p70S6K1-deficient mice, accompanied by enhanced beta-oxidation, increase in muscle AMP-activated protein kinase (AMPK), and increased life span [7], [8].

PF-4708671 is a cell-permeate p70S6K1-specific inhibitor [9]. In vitro, PF-4708671 inhibits p70S6K1 at mid nanomolar concentrations (IC_50_ of 160 nM). In a panel of ∼90 protein and lipid kinases of most closely related AGC family members, PF-4708671 was found to be highly selective for p70S6K1, being 400-fold less potent for inhibiting S6K2. Furthermore, PF-4708671 does not inhibit pyruvate dehydrogenase kinase, isozyme 1 (PDK1), which acts upstream of several AGC kinases, including p70S6K1. Among the AGC kinase family, mitogen- and stress-activated protein kinase 1 (MSK1) was found to be the next most sensitive to PF-470867, with an IC_50_ of 950 nM in vitro. However, PF-470867 did not appear to inhibit cellular MSK1 within the 1 to 10 micromolar concentration range, where it strongly inhibits p70S6K1 [9]. Due to its high specificity, PF-470867 is extensively used by studies that focus on verifying the role of p70S6K1 and mTOC1 in regulating metabolism.

Here we show that PF-4708671 activates AMPK in p70S6K 1 & 2 double knockout (DKO) immortalized mouse embryonic fibroblasts (MEF), implying AMPK activation independently of P70S6K1 inhibition. It does so directly by inhibiting mitochondrial respiratory chain Complex I. Since AMPK inhibits mTORC1 and p70S6K1 activities, its direct activation by PF-4708671 may amplify p70S6K inhibition by PF-4708671.

## Methods

### Cell culture

Immortalized mouse embryonic fibroblasts (MEF) derived from S6K double knockout or S6K wild type cells were a kind gift of Mario Pende (Inserm, Paris, France) [10] (figure S1). MEFs were cultured in DMEM (GIBCO) supplemented with 10% fetal calf serum and Pen-Strep (Biological industries, Beit HaEmek, Israel), Rapamycin and PF-4708671(Sigma-Aldrich) as indicated. A validation batch of original PF-4708671 was kindly provided by Dario Alessi (University of Dundee, Dundee, Scotland). The compound was dissolved in dimethyl sulphoxide (DMSO) and 10 mM stock solution aliquots were kept in −80°C.

### Isolation of Mitochondria

MEF mitochondria were isolated by differential centrifugation: cells were homogenized in buffer A (320 mmol/L sucrose, 5 mmol/L Tris-HCl, 2 mmol/L EGTA, pH 7.4) with a Dounce homogenizer (Teflon glass) and centrifuged for 3 min at 2,000 g to remove nuclei and cell debris. The supernatant was centrifuged for 10 min at 12,000 g at 4°C, and the pellet was re-suspended in buffer A containing 0.02% digitonin (Sigma-Aldrich) and re-centrifuged. The mitochondrial pellet was washed again twice with buffer A and kept at –80°C until use.

### Activity of mitochondrial electron transport components

Enzymatic activities of respiratory chain complexes were measured at 37_C_ by standard spectrophotometry, as previously described [11]. Briefly, Complex I was measured as rotenone-sensitive NADH-CoQ reductase, by monitoring the oxidation of NADH at 340 nm in the presence of coenzyme Q1. Complex II was measured at 600 nm by succinate-mediated phenazine methosulfate reduction of dichloroindophenol by succinate dehydrogenase (SDH). Complexes II+III were measured at 550 nm by succinate-mediated cytochrome c reduction. Complex IV (cytochrome c oxidase) was measured by the oxidation of reduced cytochrome c at 550 nm.

Citrate synthase (CS), a ubiquitous mitochondrial matrix enzyme serving as normalizer, was measured at 412 nm by the release of CoASH coupled to 50,50-dithiobis (2-nitrobenzoic) acid, in the presence of acetyl-CoA and oxaloacetate. Activities of mitochondrial respiratory chain complexes in the presence of added PF-4708671 are presented relative to respective activities in the presence of vehicle (DMSO). Protein concentration was determined by the Lowry method and calculated according to a bovine serum albumin (BSA) standard curve.

### Seahorse oxygen consumption assay

L-15medium (Beit-Haemek biological industries; http://www.bioind.com/page_14372) containing 5% FCS (Beit-Haemek biological industries) was used as the standard medium in the XF and referred to as “assay medium”. The Seahorse platform can calculate the extracellular acidification rate (ECAR) in a non-buffered medium. Thus, because L-15 is HEPES buffered extracellular acidification rate (ECAR) was not used.

MEF cells were seeded in XF 24-well cell culture microplates (Seahorse Bioscience) at 20×10^3^ cells/well (0.32 cm2) in 500 µl complete growth medium (DMEM +10% FCS) and then incubated at 37°C/5% CO_2_ for 16 h, followed by discarding the growth medium and washing the cells three times with 750 µl of assay medium. The cells were further incubated at 37°C with no CO_2_ supplementation for 4 hours to allow temperature and pH equilibration. Prior to each measurement, the XF24 Analyzer mixed the assay media for 3 min followed by 2 minutes wait to allow for oxygen pressure equilibration. Then oxygen consumption rate (OCR) was measured for 3 min. This was repeated to establish the OCR baseline. PF-4708671 or Rapamycin were injected into each well to reach the final working concentration of 2.5/5 µM and 50 nM, respectively. DMSO prepared in assay medium was injected as control. After the compounds injection, OCR measurements were made. Responses were expressed as a percentage of the baseline rate prior to compound addition. Statistical analysis was done using Seahorse analysis software using OCR area under the curve ANOVA test, as recommended by the manufacturer.

### Western blot

Cells were washed briefly in ice cold PBS, followed by adding lysis buffer (0.1M Tris pH6.8 containing 1% SDS). Lysate was incubated on ice for 30 minutes, and then centrifuged at 14,000 g at 4°C for 10 minutes. For Western blot analysis, 20 µg of protein extract was subjected to 8% or 10% SDS/PAGE and transferred to nitrocellulose membranes. After blocking in 5% non-fat milk, blots were incubated with AMPK (#2532), Phospho-AMPK (#2531), ACC (#3662), Phospho-ACC (#3661), P70S6K1 (#9202), Phospho-S6 ribosomal protein 240/244 (#5364), Phospho-S6 ribosomal protein 235/236 (#4858), beta-actin (#4967) antibodies for overnight, at 4°C. All antibodies were from Cell Signaling Technology. Blots were washed in Tris buffered saline - 0.2% Tween20 (TBS-T) three times, and incubated for one hour at room temperature with anti-Rabbit or anti-Mouse secondary antibody diluted 1∶350 in 5% no-fat milk (ImmPress peroxidase, Vector). Detection was performed using chemiluminescence reagent on microchemi digital platform (DNR bio-imaging systems). Protein concentration in extracts was determined using the BCA protein reagent (Pierce).

## Results

**Figure 1:**
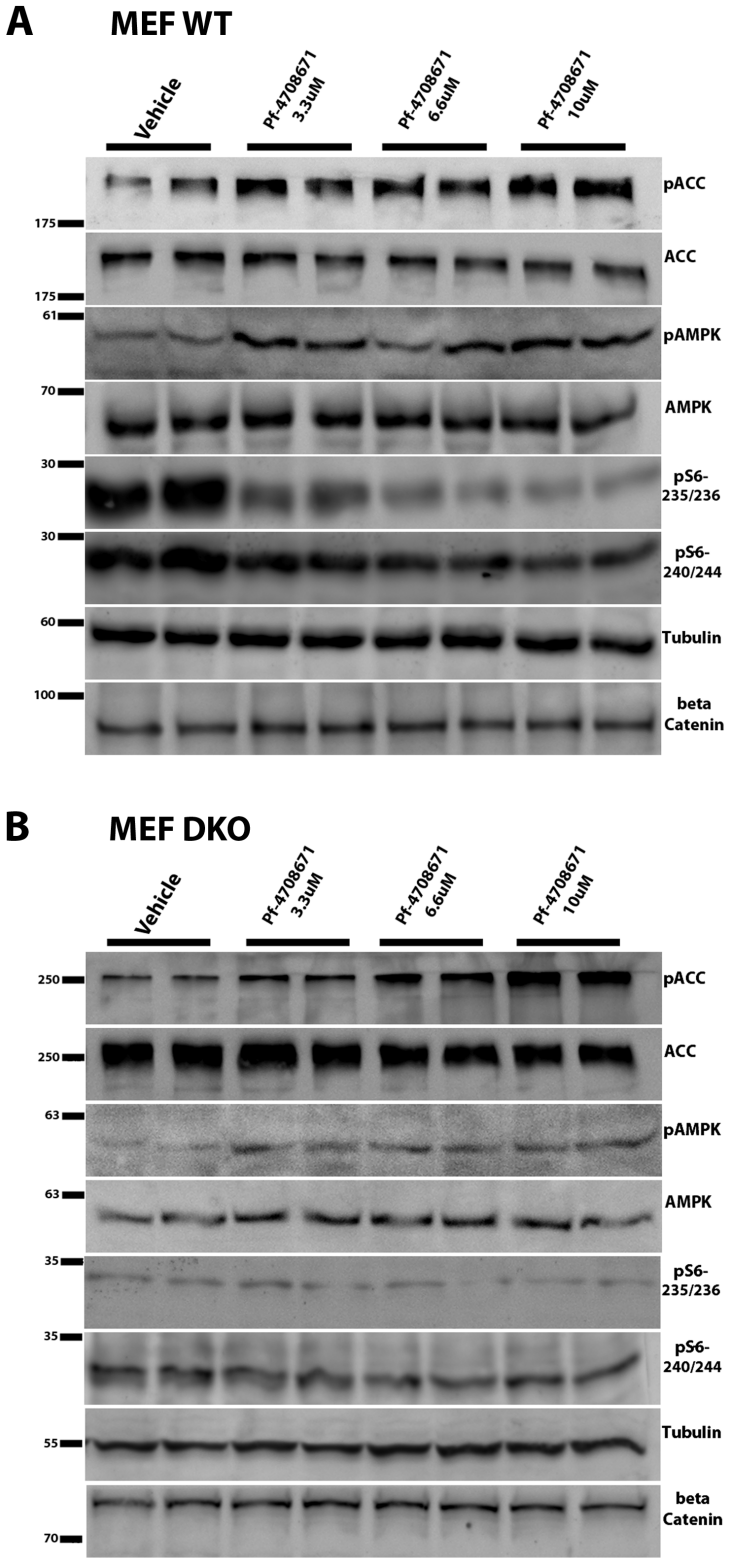
AMPK activation by PF-4708671. Wild type MEF (**A**) and p70S6K double knockout MEF (**B**) were treated with PF-4708671 as indicated. Phospho-ACC (Ser79), ACC, phospho-AMPK(Thr172), AMPK, phospho-S6 and (235/236 and 240/244 were determined by SDS-PAGE as described in Methods. Tubulin and beta-Catenin served as loading controls.

In line with previous reports [9], phosphorylation of ribosomal protein S6 (rpS6) at positions 235/236 and 240/244 was dose-dependently inhibited in wild type MEF cultured in the presence of PF-4708671 (Figure 1A and S3 for densitometry data). Concomitantly with P70S6K1 inhibition, added PF-4708671 resulted in dose-dependent phosphorylation of AMPK(Thr172) and its ACC(Ser79) downstream substrate to an extent similar to that induced by oligomycin (Figure 1A). Phosphorylation of AMPK(Thr172) and ACC(Ser79) by added PF-4708671 was similarly observed in p70S6K 1/2 double knockout MEF (S6K DKO MEF ) (Figure 1B and S3 for densitometry data), indicating that AMPK activation by PF-4708671 was p70S6K1 independent. Of note, PF-4708671 decreased the phosphorylation level of rpS6(235/236, 240/244) in S6K DKO MEF, namely, independently of p70S6K1. Furthermore, the effect of PF-4708671 is not MEF specific as other cell lines, as BT-474, a human breast cancer cell line, show elevation in ACC(Ser79) phosphorylation (Figure S2).

**Figure 2:**
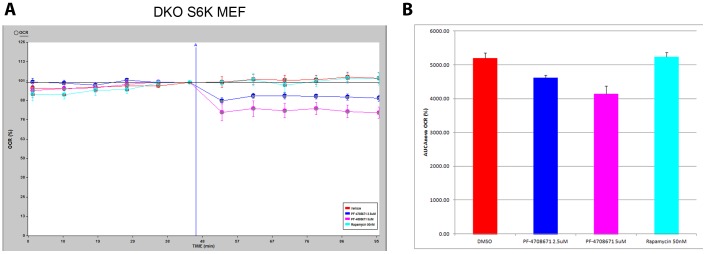
Inhibition of oxygen consumption by PF-4708671. (**A**) S6K double knockout MEF were treated with vehicle (red line); rapamycin 50 nM (turquoise); PF-4708671 2.5 µM (blue) and 5 µM (pink). Oxygen consumption rate (OCR) of was determined by the Seahorse platform as described in Methods. (**B**) ANOVA statistical analysis of the OCR area under the curve showed that after 2.5 uM or 5 uM PF-4708671 treatment the OCR was significantly decreased (p = 2.6*10^−5^ and 1.97*10^−9^, respectively; see Table S1).

Concomitantly with AMPK activation, PF-4708671 inhibited dose dependently the oxygen consumption of DKO MEF (Figure 2; and of BT-474 see Figure S2), resulting in 10 and 20% decrease by PF-4708671 concentrations of 2.5 µM and 5 µM, respectively. PF-4708671 effect in suppressing oxygen consumption rate was immediate, steady, and maintained for over one hour.

**Figure 3:**
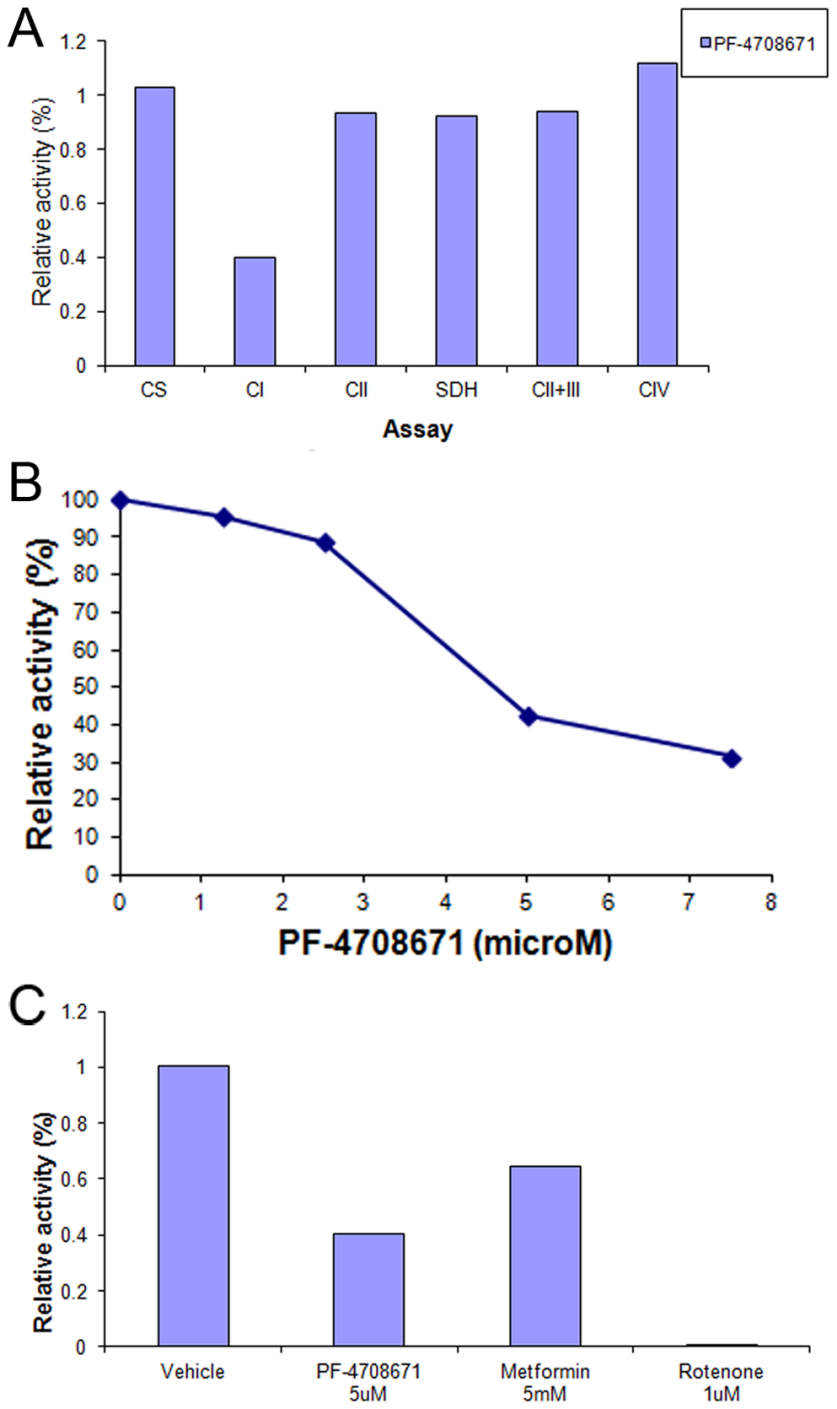
Inhibition of mitochondrial complex I by PF-4708671. MEF mitochondria were prepared as described in Methods and incubated in the presence of PF-4708671, metformin or rotenone as indicated. (**A**) Activities of citrate synthase and mitochondrial respiratory chain complexes were determined as described in Methods. Activities of mitochondrial respiratory chain complexes in the presence of added PF-4708671 are presented relative to respective activities in the presence of vehicle. (**B**) Relative activity of mitochondrial Complex I in the presence of increasing PF-4708671 concentrations as indicated. (**C**) Relative activity of mitochondrial Complex I in the presence of PF-4708671, metformin or rotenone as indicated. Activities of mitochondrial respiratory chain complexes are presented relative to (DMSO).

Inhibition of oxygen consumption by PF-4708671 was further pursued by studying PF-4708671 effects in isolated mitochondria. Mitochondrial citrate synthase, Complex II, succinate dehydrogenase, Complex II+III, and complex IV activities were unaffected by PF-4708671 (Figure 3A). However, mitochondrial respiratory chain Complex I activity was inhibited in a concentration dependent manner, with an IC50 of 5.2 µM (Figure 3A–B). Complex I inhibition was highly statistically significant between DMSO and 7.5 µM PF-4708671 (t-Test 1.4*10^−6^; Table S1). Inhibition of mitochondrial Complex I by 5 µM PF-4708671 was comparable to that of 5 mM metformin. Mitochondrial Complex I activity was similarly inhibited by an original batch of PF-4708671 (data not shown), implying that inhibition was inherently due to PF-4708671, rather than an impurity in the commercial sample.

## Discussion

The present study describes activation of AMPK by PF-4708671, a novel cell-permeable P70S6K1 inhibitor. AMPK activation by PF-4708671 is independent of p70S6K1, and may be ascribed to mitochondrial Complex I inhibition. Thus, AMPK activation and suppression of oxygen consumption by PF-4708671 were both observed in p70S6K null cells. The calculated IC_50_ of PF-4708671 in inhibiting mitochondrial Complex I was ∼5 µM, namely, within the working concentration range of 3–10 µM used to effectively block p70S6K1 [9].

p70S6K1 is activated by phosphorylation of its Thr389 by mTORC1 [3]. However, mTORC1 is one of the downstream targets of AMPK, being inhibited by AMPK via multiple mechanisms [12], [13]. Hence, inhibition of p70S6K1 by PF-4708671 may be a result of both, direct inhibition of its kinase activity complemented by inhibition of its upstream mTORC1 activator by PF-4708671-activated AMPK. In line with that, AMPK activation by PF-4708671 may partially account for PF-4708671 suppression of phospho-rpS6(235/235, 240/244), independently of P70S6K1 (Fig 1B). Indeed, in addition to p70S6K, rpS6 may directly be phosphorylated by p90S6K (RSK) [6], [14] that may cross talk with AMPK, independently of p70S6K1.

In conclusion, direct activation of AMPK by PF-4708671, combined with inhibition of P70S6K1 activity, may open new prospects for PF-4708671 in modifying mTORC1 and p70S6K1 control of metabolism, protein synthesis, autophagy, and proliferation.

## Supporting Information

Figure S1 **DKO and WT MEFs comparison. p70S6K double knockout MEF show lower levels of phospho-rpS6.** Wild type MEF and p70S6K double knockout MEF were grown in complete medium. p70S6K1 and phospho-S6 (235/236 and 240/244) were determined by SDS-PAGE as described in Methods. Tubulin served as loading control.
(TIF)

Figure S2 **BT-474 show pACC elevation due to PF-4708671.** BT-474 were treated with PF-4708671 as indicated. Phospho-ACC (Ser79), phospho- AKT (Ser473), AKT, and phospho-S6 (235/236 and 240/244) were determined by SDS-PAGE as described in Methods. Tubulin served as loading control.
(TIF)

Figure S3 **Densitometry of** **Figure 1** **. AMPK activation by PF-4708671.** Wild type MEF **(A)** and p70S6K double knockout MEF **(B)** were treated with PF-4708671 as indicated. Densitometry of Phospho-ACC (Ser79), ACC, phospho-AMPK(Thr172), AMPK, and phospho-S6 (235/236 and 240/244) were determined by chemiluminescence as described in Methods. ACC, AMPK or Tubulin served as loading controls, as indicated in the figures.
(TIF)

Figure S4 **Inhibition of BT-474 oxygen consumption by PF-4708671.** BT-474 were treated with vehicle (turquoise line) or 3 µM of PF-4708671(pink). Oxygen consumption rate (OCR) of was determined by the Seahorse platform as described in Methods. As shown before, PF-4708671 effect in suppressing oxygen consumption rate was immediate, steady, and maintained for over one hour.
(TIF)

Table S1 **Seahorse platform and mitochondrial assays statistics.** ANOVA statistical analysis of the OCR area under the curve showed that after 2.5 uM or 5 uM PF-4708671 treatment the OCR was significantly decreased (manufacturer output), and mitochondrial assay statistics.
(XLSX)
