# Supplementary figures and images for: PF-4708671 Activates AMPK Independently of p70S6K1 Inhibition
Source: PLoS One. 2014 Sep 9;9(9):e107364. doi: 10.1371/journal.pone.0107364 (PMC4159345; doi:10.1371/journal.pone.0107364)

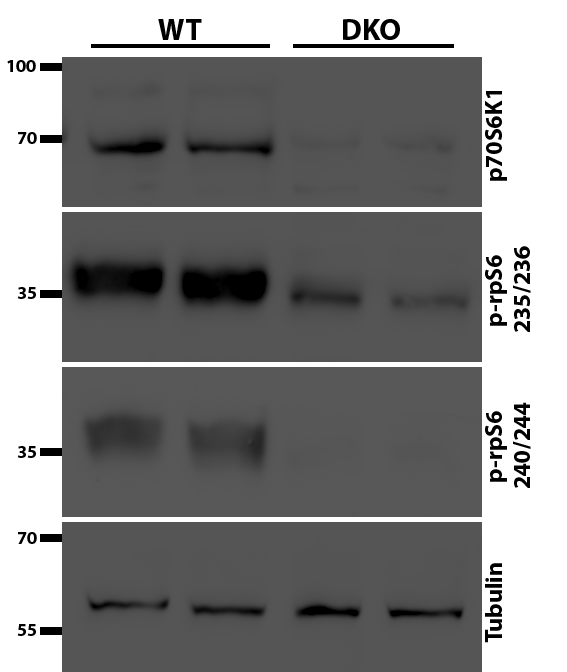

Supplement: Figure S1 — DKO and WT MEFs comparison. p70S6K double knockout MEF show lower levels of phospho-rpS6. Wild type MEF and p70S6K double knockout MEF were grown in complete medium. p70S6K1 and phospho-S6 (235/236 and 240/244) were determined by SDS-PAGE as described in Methods. Tubulin served as loading control. (TIF) [file pone.0107364.s001.tif]

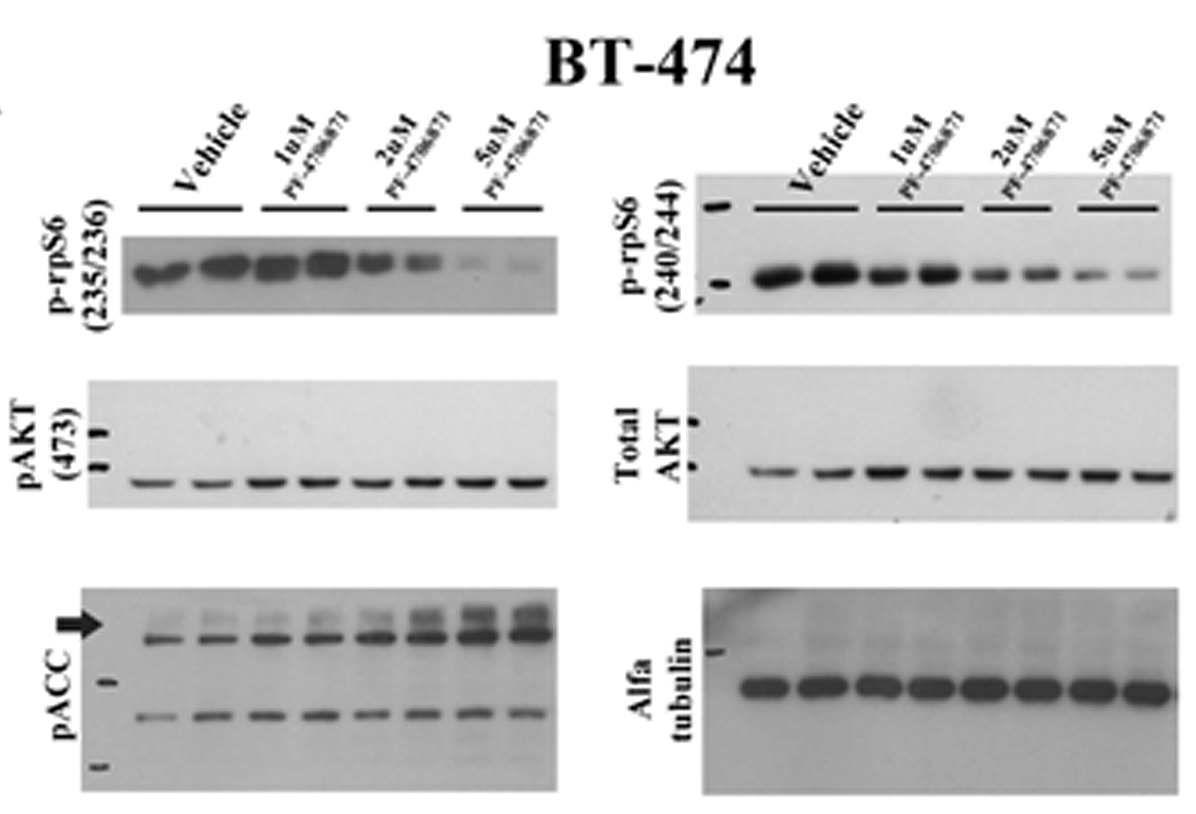

Supplement: Figure S2 — BT-474 show pACC elevation due to PF-4708671. BT-474 were treated with PF-4708671 as indicated. Phospho-ACC (Ser79), phospho- AKT (Ser473), AKT, and phospho-S6 (235/236 and 240/244) were determined by SDS-PAGE as described in Methods. Tubulin served as loading control. (TIF) [file pone.0107364.s002.tif]

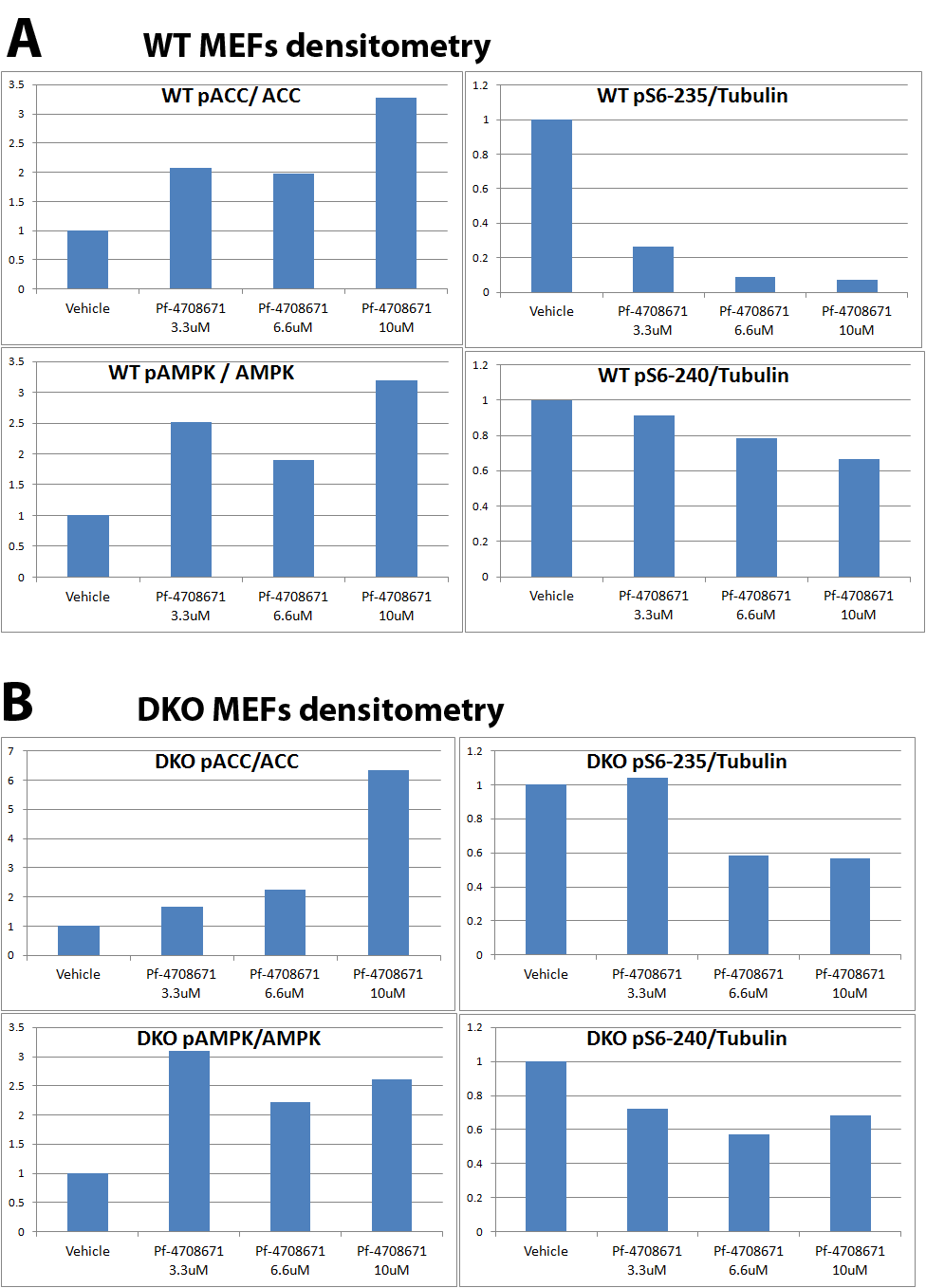

Supplement: Figure S3 — Densitometry of Figure 1 . AMPK activation by PF-4708671. Wild type MEF (A) and p70S6K double knockout MEF (B) were treated with PF-4708671 as indicated. Densitometry of Phospho-ACC (Ser79), ACC, phospho-AMPK(Thr172), AMPK, and phospho-S6 (235/236 and 240/244) were determined by chemiluminescence as described in Methods. ACC, AMPK or Tubulin served as loading controls, as indicated in the figures. (TIF) [file pone.0107364.s003.tif]

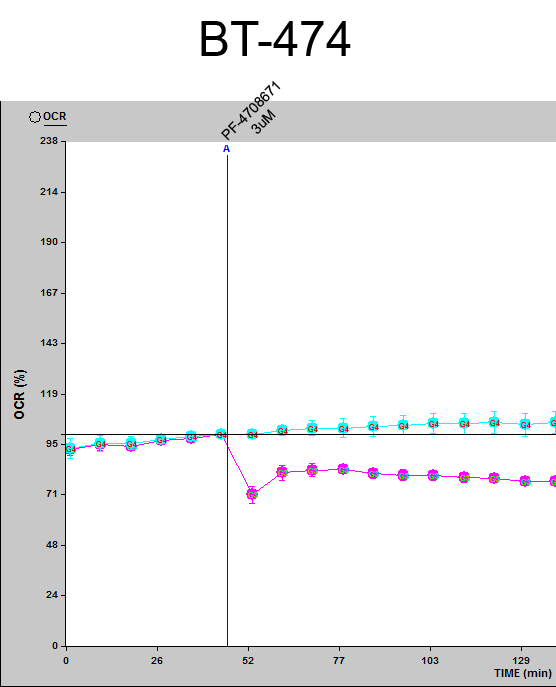

Supplement: Figure S4 — Inhibition of BT-474 oxygen consumption by PF-4708671. BT-474 were treated with vehicle (turquoise line) or 3 µM of PF-4708671(pink). Oxygen consumption rate (OCR) of was determined by the Seahorse platform as described in Methods. As shown before, PF-4708671 effect in suppressing oxygen consumption rate was immediate, steady, and maintained for over one hour. (TIF) [file pone.0107364.s004.tif]
